# Nuclear Nox4 Role in Stemness Power of Human Amniotic Fluid Stem Cells

**DOI:** 10.1155/2015/101304

**Published:** 2015-07-26

**Authors:** Tullia Maraldi, Marianna Guida, Manuela Zavatti, Elisa Resca, Laura Bertoni, Giovanni B. La Sala, Anto De Pol

**Affiliations:** ^1^Department of Surgical, Medical, Dental and Morphological Sciences with Interest in Transplant, Oncology and Regenerative Medicine, University of Modena and Reggio Emilia, Via Del Pozzo 71, 41100 Modena, Italy; ^2^EURAC Research, Center for Biomedicine, Via Galvani 31, 39100 Bolzano, Italy; ^3^Unit of Obstetrics & Gynecology, IRCCS-Arcispedale Santa Maria Nuova, Viale Umberto I 50, 42123 Reggio Emilia, Italy; ^4^University of Modena e Reggio Emilia, Viale A. Allegri 9, 42121 Reggio Emilia, Italy

## Abstract

Human amniotic fluid stem cells (AFSC) are an attractive source for cell therapy due to their multilineage differentiation potential and accessibility advantages. However the clinical application of human stem cells largely depends on their capacity to expand *in vitro*, since there is an extensive donor-to-donor heterogeneity. Reactive oxygen species (ROS) and cellular oxidative stress are involved in many physiological and pathophysiological processes of stem cells, including pluripotency, proliferation, differentiation, and stress resistance. The mode of action of ROS is also dependent on the localization of their target molecules. Thus, the modifications induced by ROS can be separated depending on the cellular compartments they affect. NAD(P)H oxidase family, particularly Nox4, has been known to produce ROS in the nucleus. In the present study we show that Nox4 nuclear expression (nNox4) depends on the donor and it correlates with the expression of transcription factors involved in stemness regulation, such as Oct4, SSEA-4, and Sox2. Moreover nNox4 is linked with the nuclear localization of redox sensitive transcription factors, as Nrf2 and NF-*κ*B, and with the differentiation potential. Taken together, these results suggest that nNox4 regulation may have important effects in stem cell capability through modulation of transcription factors and DNA damage.

## 1. Introduction

Numerous studies have demonstrated that the MSC populations exhibit donor-to-donor heterogeneity. This fact could be attributed to several factors, including the methods used to culture, select, and expand the population and the age of the donor [1].

About amniotic fluid stem cells (AFSC), the harvesting protocol is well established in the clinical practice as well as the selection method, based on the c-Kit surface marker expression [2]. Moreover the donor age range has to be considered quite restricted since the sample is usually obtained in clinical practice for cytogenetic analysis between the 16th week and the 20th week of pregnancy. However, as well as other MSCs [1], AFSC could display heterogeneity among the donors.

Regulation of ROS has a vital role in maintaining the “stemness” and the differentiation potential of the stem cells, as well as in the progression of stem-cell-associated diseases [3]. ROS-mediated proliferation and senescence in stem/progenitor cells may be determined by the amount, duration, and location of ROS generation, which activates specific redox-signaling pathways [4]. In fact redox changes in different areas and resulting changes in ROS levels may represent an important mechanism of intracellular communication between different cellular compartments [5]. The nucleus itself contains a number of proteins with oxidizable thiols that are essential for transcription, chromatin stability, and nuclear protein import and export, as well as DNA replication and repair [5]. Several transcription factors have been thought to be involved in the redox-dependent modulation of gene expression [5].

Recent advances indicate that the participation of ROS-producing nicotinamide adenine dinucleotide phosphate reduced oxidase (NADPH, Nox) system is an important trigger for differentiating ESCs toward the cardiomyocyte lineage [6–10]. Nox4 plays an important role in the differentiation of mouse ESCs toward the smooth muscle cell (SMC) lineage when translocating to the nucleus and generating H_2_O_2_ [11]. In fact the subcellular localization of Nox4 is likely to be especially important, given its constitutive activity, unlike isoforms, such as Nox1 or Nox2, that require agonist activation. Nox4 has been reported to be variably present in the endoplasmic reticulum [12, 13], mitochondria [14], cytoskeleton [15], plasma membrane [16], and nucleus [17] in different cell types. Recently we demonstrated that Nox4 can be detected in nuclei of human AFSC, depending on the cell metabolism status [18].

It is interesting to better understand how ROS homeostasis is an important modulator in stem cell self-renewal and differentiation. Certain proteins can act as “redox sensors” due to the redox modifications of their cysteine residues, which are critically important in the control of protein function. Signaling molecules such as FoxOs, APE1/Ref-1, Nrf2, ATM, HIFs, NF-*κ*B, p38, and p53 are subjected to redox modifications and could be involved in the regulation of stem cell self-renewal and differentiation [19].

The aim of this study was to assess whether nuclear Nox4-generated ROS can modulate the presence and the localization in nuclear domain of transcription factors crucial for stemness capability. For this purpose we performed confocal analysis of immunofluorescence experiments and coimmunoprecipitation assays. Furthermore we investigated whether the different nuclear Nox4 (nNox4) presence, observed among the AFSC samples, was correlated with the expression of typical stem cell markers and the differentiation potential. These data indicate that nNox4 derived ROS are involved in AFSC stemness regulation and could be considered as marker of stem potential.

## 2. Materials and Methods

### 2.1. Cell Culture

Amniocentesis samples (6 backup flasks obtained from different donors) were provided by the Laboratorio di Genetica, Ospedale Santa Maria Nuova (Reggio Emilia, Italy). All samples were collected with the informed consent of the patients (mother's age ≥ 35) according to Italian law and the Ethical Committee guideline.

Human AFSC (AFSC) were isolated as previously described by De Coppi et al. [2]. Human amniocentesis cultures were harvested by trypsinization and subjected to c-Kit immunoselection using MACS technology (Miltenyi Biotec, Germany). AFSC were subcultured routinely at 1 : 3 dilution and were not allowed to expand beyond the 70% of confluence. AFSC were grown in a culture medium (*α*MEM supplemented with 20% fetal bovine serum (FBS), 2 mM L-glutamine, 100 U/mL penicillin, and 100 *μ*g/mL streptomycin) (all reagents from EuroClone Spa, Italy) at 37°C and 5% CO_2_ [20].

### 2.2. Nox4 Silencing

Retroviral supernatants were produced according to HuSH shRNA plasmid panels (29-mer) application guide; AM12 cells were transfected with an empty vector (pRS vector, TR20003), a scrambled vector (HuSH 29-mer noneffective pRS vector, TR30012), and four NOX4 gene specific shRNA expression pRS vectors (TI311637, TI311638, TI311639, and TI311640) for 48 h [21]. Retroviral supernatants were then centrifuged at 2000 ×g for 5 minutes and used for target cells (AFSC) infection. Where indicated, cells were infected with NOX4 shRNA retroviral vectors, empty vector, or scrambled vector. Forty-eight hours after infection, cells were exposed to 2 *μ*g/mL puromycin (Sigma-Aldrich, St. Louis, MO, USA) for 24 hours and subjected to evaluation of Nox4 expression by western blotting and confocal analysis and detection of intracellular ROS levels.

### 2.3. Differentiation Protocols

Osteogenic differentiation was obtained maintaining cells for 3 weeks at 37°C and 5% CO_2_ in osteogenic medium: culture medium supplemented with 100 nM dexamethasone, 10 mM *β*-glycerophosphate, and 50 *μ*g/mL ascorbic acid-2-phosphate (Sigma-Aldrich, St. Louis, MO, USA). Coverslips were then stained with alizarin red S staining for light microscopic observation.

Chondrogenic differentiation: cells were cultured as a monolayer using a medium containing DMEM high glucose, 100 nM dexamethasone and 10 ng/mL TGF *β*1 (Sigma-Aldrich, St. Louis, USA), 10 *μ*M 2P-ascorbic acid, 1% v/v sodium pyruvate (Invitrogen, Italy), and 50 mg/mL ITS premix (BD, Franklin Lakes, NJ, USA) for 3 weeks.

Neural differentiation protocol [22]: cells were seeded at 60% confluence and maintained in neural differentiation medium (culture medium supplemented with 10% FBS and 20 *μ*M retinoic acid (RA) in dimethyl sulfoxide (DMSO), both from Sigma-Aldrich, St. Louis, MO, USA) for up to 4 weeks at 37°C and 5% CO_2_.

### 2.4. Preparation of Cell Extracts

Cell extracts were obtained as described by Maraldi et al. [23]. Briefly, subconfluent cells were extracted by addition of AT lysis buffer (20 mM Tris-Cl, pH 7.0; 1% Nonidet P-40; 150 mM NaCl; 10% glycerol; 10 mM EDTA; 20 mM NaF; 5 mM sodium pyrophosphate; and 1 mM Na_3_VO_4_) and freshly added Sigma-Aldrich protease inhibitor cocktail at 4°C for 30 min. Lysates were sonicated, cleared by centrifugation, and immediately boiled in SDS sample buffer or used for immunoprecipitation experiments, as described below.

### 2.5. Immunoprecipitation and Electrophoresis

Immunoprecipitation was performed as reported by Cenni et al. [24]. Equal amounts of precleared lysates (pcl), whose protein concentration was determined by the Bradford method, were incubated overnight with rabbit anti-Nox4 (Novus Biologicals, CO, USA) and mouse anti-sc-35 (Sigma-Aldrich) (3 *μ*g all). Then the two samples were treated with 30 *μ*L of 50% (v/v) of protein A/G agarose slurry (GE Healthcare Bio-sciences, Uppsala, Sweden) at 4°C with gentle rocking for 1 h. Pellets were washed twice with 20 mM Tris-Cl, pH 7.0; 1% Nonidet P-40; 150 mM NaCl; 10% glycerol; 10 mM EDTA; 20 mM NaF; and 5 mM sodium pyrophosphate, once with 10 mM Tris-Cl, pH 7.4, boiled in SDS sample buffer, and centrifuged. Supernatants were loaded onto SDS-polyacrylamide gel, blotted on Immobilon-P membranes (Millipore, Waltham, MA, USA), processed by western blot with the indicated antibodies and detected by Supersignal substrate chemiluminescence detection kit (Pierce, Rockford, IL, USA). Signal quantification was obtained by chemiluminescence detection on a Kodak Image Station 440CF and the analysis with the Kodak 1D Image software.

### 2.6. Nuclei Purification

Human AFSC nuclei were purified as reported by Cenni et al. [25]. Briefly, 400 *μ*L of nuclear isolation buffer (10 mM Tris-HCl, pH 7.8, 1% Nonidet P-40, 10 mM *β*-mercaptoethanol, 0.5 mM phenylmethylsulfonyl fluoride, 1 *μ*g/mL aprotinin and leupeptin, and 5 mM NaF) was added to 5 × 10^6^ cells for 8 min on ice. Milli-Q water (400 *μ*L) was then added to swell cells for 3 min. Cells were sheared by passages through a 22-gauge needle. Nuclei were recovered by centrifugation at 400 ×g at 4°C for 6 min and washed once in 400 *μ*L of washing buffer (10 mM Tris-HCl, pH 7.4, and 2 mM MgCl_2_, plus inhibitors as described earlier in the text). Supernatants (containing the cytosolic fractions) were further centrifuged for 30 min at 4000 ×g. Isolated nuclear and cytoplasmic extracts were finally lysed in AT lysis buffer, sonicated, and cleared by centrifugation.

### 2.7. Western Blot

The protocols of the western blot were performed as described by Hanson et al. [26].

Protein extracts, quantified by a Bradford Protein Assay (Bio-Rad Laboratories, CA, USA), underwent SDS-polyacrylamide gel electrophoresis and were transferred to Immobilon-P membranes. The following antibodies were used: rabbit anti-NF-*κ*B, rabbit anti-*β*catenin, goat anti-matrin3, goat anti-actin (Santa Cruz Biotechnology, Santa Cruz, CA, USA) diluted 1 : 500; rabbit anti-cyclin E2, cyclin D1, cyclin B1, p21, Pmyt1, Oct4, and mouse anti-cyclin A1, and SSEA-4 (Cell Signalling Technology, Beverly, MA, USA), mouse anti-tubulin, and mouse anti-sc-35 (Sigma-Aldrich St. Louis, MO, USA), rabbit anti-Nrf2 (Abcam, Cambridge, UK), rabbit anti-Nox4 (Novus Biologicals, CO, USA), and mouse anti-pH2A (Ser139), mouse anti-CD90 and anti-CD105 (Millipore, Billerica, MA, USA) rabbit anti-CD73 (Genetex, Irvine, CA, USA), diluted 1 : 1000; peroxidase-labelled anti-rabbit, mouse, and goat secondary antibodies diluted 1 : 3000 (Pierce Antibodies, Thermo Scientific; Rockford, IL, USA). Ab dilution was performed in TBS-T pH 7.6 containing 3% BSA. The membranes were visualized using Supersignal substrate chemiluminescence detection kit (Pierce, Rockford, IL, USA). Anti-actin antibody was used as control of protein loading.

### 2.8. Senescence Assay

Senescent cells were visualized in 45 days cultures with the Senescence *β*-Galactosidase Staining Kit (Cell Signalling Technology, Beverly, MA, USA) following the manufacturer's instructions. This test is designed to detect *β*-galactosidase activity at pH 6, a known characteristic of senescent cells not found in presenescent, quiescent, or immortal cells.

### 2.9. Confocal Microscopy

Undifferentiated AFSC were fixed for 20 min in 4% ice-cold paraformaldehyde and then permeabilized with 0.1% Triton X-100 in ice-cold phosphate-buffered saline (PBS) for 5 min. Permeabilized samples were then blocked with 3% of bovine serum albumin (BSA) in PBS for 30 min at room temperature (RT) and incubated with primary antibodies (Ab). Mouse anti-sc-35 and mouse anti-glial fibrillary acidic protein (GFAP) (Sigma-Aldrich, St. Louis, MO, USA), rabbit anti-human Collagen type II (Genetex, Irvine, CA, USA), rabbit anti-coilin (Abcam, Cambridge, UK), goat anti-aggrecan, rabbit anti-Nox4, rabbit anti-Oct4, goat anti-Foxo1, goat anti-Sox2 (Santa Cruz Biotechnology, Santa Cruz, CA, USA) (diluted 1 : 50), mouse anti-Oct4 (Millipore Billerica, MA, USA), mouse anti-*β*tubulin III (Cell Signalling Technology, Beverly, MA, USA), and mouse anti-pH2A (Ser139) (Millipore, Billerica, MA, USA) (diluted 1 : 100), in PBS containing 3% BSA for 1 h at RT, were used as primary antibodies (Ab). Secondary Ab were diluted 1 : 200 in PBS containing 3% BSA (goat anti-mouse Alexa 647, goat anti-rabbit Alexa 488, and donkey anti-goat Alexa 488). After washing in PBS, samples were stained with 1 *μ*g/mL DAPI in H_2_O for 1 min and then were mounted with antifading medium (0.21 M DABCO and 90% glycerol in 0.02 M Tris, pH 8.0). Negative controls consisted of samples not incubated with the primary antibody but only with the secondary antibody.

In the case of a double staining with sc-35 antibody and, for example, Nox4, we performed a first incubation with anti-Nox4 overnight and then, separately, 1 h of incubation for anti-sc-35, in order to avoid unspecific antibodies interactions.

Confocal imaging was performed using a Nikon A1 confocal laser scanning microscope as previously described [27].

Spectral analysis was performed to exclude overlapping between two signals or the influence of autofluorescence background on the fluorochrome signals, as previously shown [28]. The confocal serial sections were processed with ImageJ software to obtain three-dimensional projections, as previously described [29]. The image rendering was performed using Adobe Photoshop software.

### 2.10. Nuclear ROS Imaging

Nuclear ROS were detected with nuclear-localized fluorescent probe for H_2_O_2_, nuclear peroxy emerald 1 (NucPE1) [30–33]. For all experiments, 5 *μ*M solutions of NucPE1 (from 5 mM stocks in DMSO) were made in PBS/glucose. The cells were then kept in an incubator (37°C, 5% CO_2_) during the course of all experiments. The probe was incubated for total of 30 min.

Confocal fluorescence imaging studies were performed with a Nikon A1 confocal laser scanning microscope. Excitation of NucPE1-loaded cells at 488 nm was carried out with an Ar laser and emission was collected at 535 nm. All images in an experiment were collected simultaneously using identical microscope settings. Image analysis was performed in ImageJ.

### 2.11. Statistical Analysis

*In vitro* experiments were performed in triplicate. For quantitative comparisons, values were expressed as mean ± SD (standard deviation) based on triplicate analysis for each sample. To test the significance of observed differences among the study groups, one way analysis of variance (ANOVA) test with the post-hoc Bonferroni correction was applied. A *P* value of <0.05 was considered to be statistically significant.

## 3. Results

### 3.1. Nox4 into the Nucleus of AFSC

**Figure 1:**
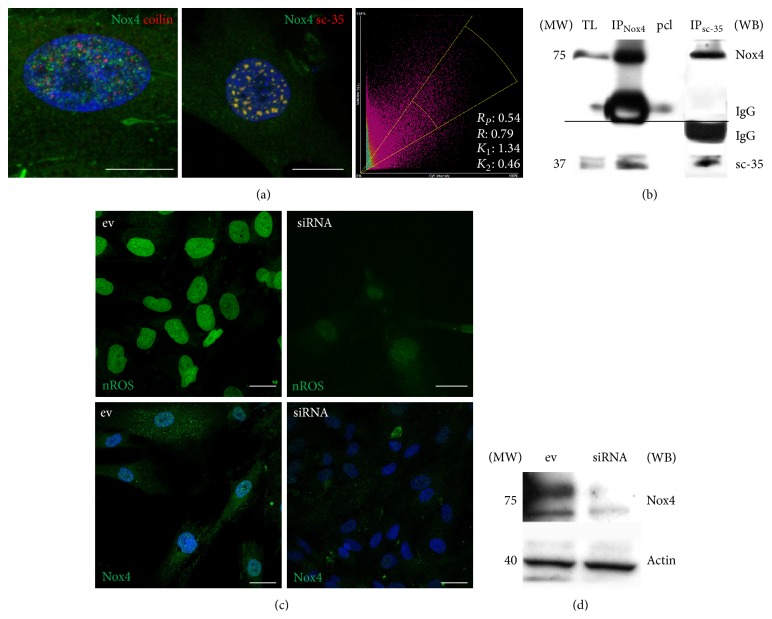
Nox4 nuclear localization and interaction in AFSC. (a) Representative images showing superimposing between DAPI (blue), Nox4 (green), and coilin (red) or sc-35 (red). Colocalization graph reporting Pearson's and overlap coefficients. Scale bar: 10 *μ*m. (b) Total lysates (TL) were immunoprecipitated with sc-35 antibody and then revealed with anti-sc-35 and anti-Nox4 (right) or were immunoprecipitated with anti-Nox4 and then revealed with anti-Nox4 and anti-sc-35 (left). Signals of preclearing sample (pcl) are shown in the middle line. (c) First line: representative images showing staining with nuclear ROS probe (nuclear peroxy emerald 1) of AFSC treated or not treated with shRNA. Second line: Nox4 signal (green) in the same samples. Scale bar: 10 *μ*m. (d) Western blot revealed anti-Nox4 of AFSC treated with empty vector (ev) or siRNA TI311640, the best silencing vector among the 4 reported in Materials and Methods section. All presented data are representative of three independent experiments.

Recently we have shown that, by using antibodies from Santa Cruz, Abcam, or Novus, we can see a Nox4 signal mostly localized inside the nuclei of AFSC [18]. In particular AFSC expressing Nox4 into the nucleus show a spot distribution, a punctate pattern similar to the one observed in nuclear domains, such as speckles or Cajal bodies. In order to test whether nuclear Nox4 (nNox4) resides inside nuclear domains, colocalization assays were performed using antibodies directed to sc-35, a speckle marker, or coilin, a Cajal bodies marker. Confocal analysis (Figure 1(a)) of double staining with anti-Nox4 (green) and anti-sc35 (red) or anti-coilin (red) demonstrates that Nox4 interacts with domain of nuclear speckles, rather than with Cajal bodies, as shown by values of the Pearson's correlation coefficient (*Rp*) and overlap coefficient (*R*), which provides information about the similarity of shape between the two patterns (Nox4 and sc-35). The value for correlation* R* can ranges from −1 to 1, and thus a value of 1 would mean that the patterns are perfectly similar, while a value of −1 would mean that the patterns are perfectly opposite. An overlap coefficient around 0.8 indicates a very good colocalization of the two signals.

To demonstrate that this localization means also a direct interaction of these proteins, coimmunoprecipitation experiments (IP for anti-sc35 and IP for anti-Nox4) were performed and show that Nox4 interacts with domain of nuclear speckles (Figure 1(b)).

In order to investigate the NADPH oxidase activity inside the nuclei, we used a nuclear selective probe for H_2_O_2_, nuclear peroxy emerald 1 (Figure 1(c)). Immunofluorescence assay (Figure 1(c)) shows that the decrease in Nox4 expression, demonstrated by western blot (Figure 1(d)), occurs both in cytoplasmic and nuclear compartments. Overall, AFSC cells, treated with siRNA, show a significant decrease in nuclear ROS level.

**Figure 2:**
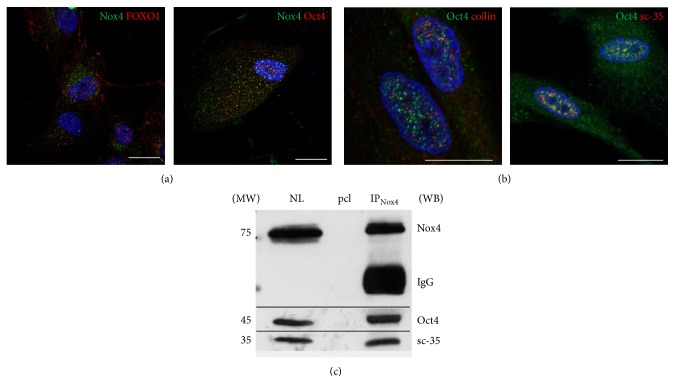
Nox4 interaction with transcription factors in nuclei of AFSC. (a) Representative images showing superimposing between DAPI (blue), Nox4 (green), and FoxO1 (red) or Oct4 (red). (b) Representative images showing superimposing between DAPI (blue), Oct4 (green), and coilin (red) or sc-35 (red). Scale bar: 10 *μ*m. (c) Western blot analysis of nuclear lysate (NL) and immunoprecipitation experiment of NL with Nox4 antibody then revealed with anti-Oct4 and anti-sc-35. Signals of preclearing sample (pcl) are shown in the middle line. Presented data are representative of three independent experiments.

Forkhead Box O (FoxO) transcription factors act in adult stem cells to preserve their regenerative potential. FoxO1 is essential for the maintenance of human ESC pluripotency. This function is probably mediated through direct control exerted by FoxO1 of Oct4 and Sox2 gene expression through occupation and activation of their respective promoters [34]. The cellular distribution of FoxO1 in AFSC is both in the cytosol and in the nucleus, but the Nox4 signal matches only in the cytosol, as shown in Figure 2(a). Otherwise, the pluripotent stem cell marker Oct4 colocalizes in some spots with nNox4 staining into the nucleus (Figure 2(a)). Interestingly Oct4 is detectable in speckle domains, as shown by labeling with sc-35 (Figure 2(b)) and coimmunoprecipitation assay (Figure 2(c)). The signal of coilin, a Cajal bodies marker, does not match with the Oct4 one (Figure 2(b)).

### 3.2. AFSC Heterogeneity and nNox4

**Figure 3:**
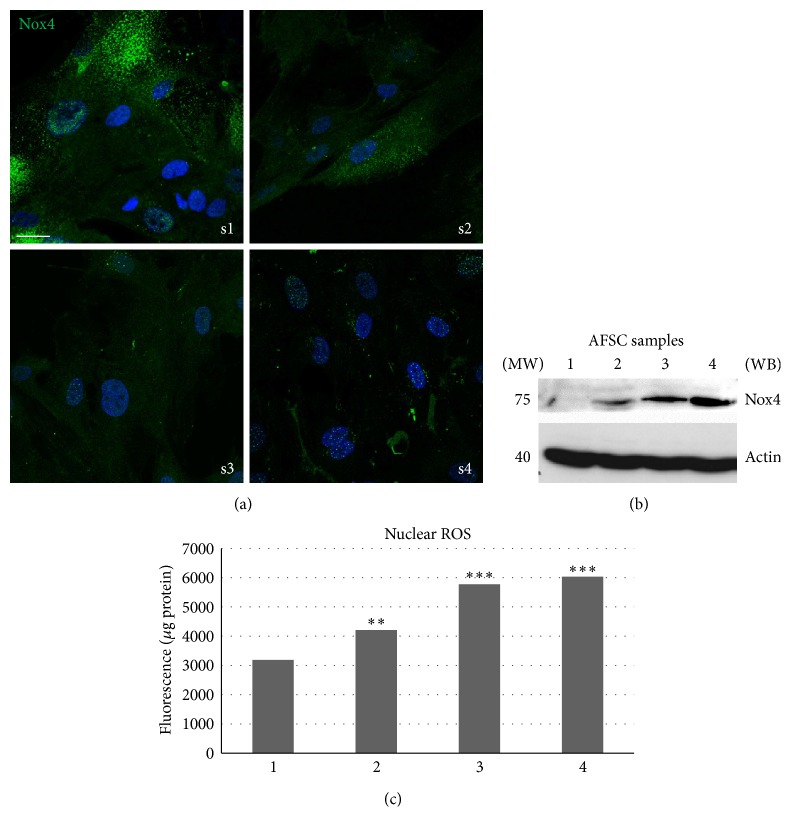
Effect of donor heterogeneity on Nox4 localization and nuclear ROS production. (a) Representative images showing superimposing between DAPI (blue) and Nox4 (green) signals of 4 different AFSC cultures. Scale bar: 10 *μ*m. (b) Representative images of western blot analysis of nuclei of samples 1–4 of AFSC revealed with Nox4. Actin detection was performed in order to show the amount of protein loaded in each line. Presented data are representative of three independent experiments. (c) Representative graph showing fluorescence obtained with nuclear ROS probe (nuclear peroxy emerald 1) normalized to protein content of AFSC samples. ^***^ *P* < 0.0001; ^***^ *P* < 0.01 significantly different from sample 1.

Stem cells isolated from different amniotic fluids (1–6 collected samples) exhibit different behaviors, proliferation rates. In the figures only the most representative 4 were shown.  Figure 3(a) shows images, representative of 4 of the 6 samples, related to the different cell distribution of Nox4. It is evident that in s1 sample Nox4 is more expressed and it is detectable mostly in the cytosol. Conversely, sample s4, the slowest one, shows a Nox4 localization into the nuclei, while the cytosolic expression is low. This evidence is confirmed by western blot analysis of Nox4 in nuclear extracts, as shown in Figure 3(b). Moreover, the use of nuclear ROS probe demonstrates that the production of ROS in the nuclei significantly increases from s1 to s4 donor (Figure 3(c)).

**Figure 4:**
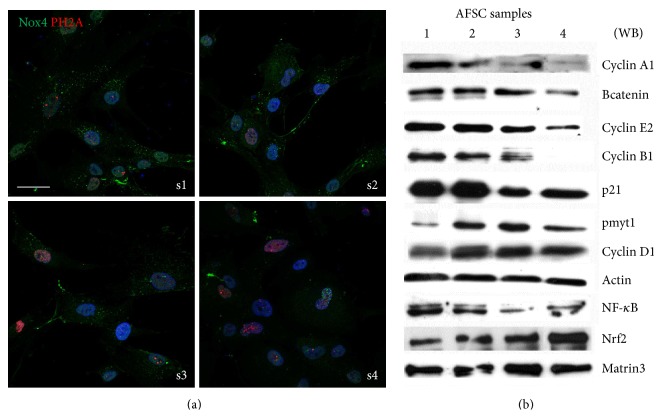
AFSC samples heterogeneity in DNA damage, senescence, and cell cycle. (a) Representative images showing superimposing between DAPI (blue), Nox4 (green), and PH2A (red) signals of samples 1 to 4 of AFSC. (b) Representative images of total lysates of AFSC samples 1–4 separated by SDS-PAGE. Western blot was then performed with the indicated antibodies. Actin detection was performed in order to show the amount of protein loaded in each line. The analysis for NF*κ*B and Nrf2 was performed on nuclear lysates and matrin3 detection was performed in order to show the amount of nuclear protein loaded in each line. Presented data are representative of three independent experiments.

Since ROS can cause DNA damage, we tested the phosphorylation level of H2AX while it is crucial to determine whether cells will survive after DNA damage [35]. As expected looking at nuclear H2A foci, we found that, compared to s1 and s2, s3 and s4 samples exhibit a huge status of H2A phosphorylation (Figure 4(a)). The double staining for Nox4 and pH2AX, even if not in all the nuclei, can suggest that nNox4-generated ROS can induce nuclear DNA damage.

In parallel, we looked for senescence marker, *β*-galactosidase activity (data not shown), but only a not significant increase can be noticed in the sample 4.

Indeed, regarding the proliferation rate, the faster sample (s1) cultured* in vitro* reaches confluence every 48 h, while the slowest one (s4), seeded at the same density, spends more than 3 days. A deeper analysis of the cell cycle is reported below (Figure 4(b)). The positivity for c-Kit in the selected population is around 98% for all the samples (data not shown).

In order to investigate cell cycle check points, we analyzed the expression of different cyclins and other related proteins (Figure 4(b)). Cyclins A1, B1, and E2, usually upregulated in proliferating cells, decrease passing from s1 to s4, as well as p21 and *β*-catenin. On the other hand, pmyt1 and cyclin D1 increase, since they are expressed during G_0_/G_1_ phase, confirming the low rate of growth of these samples (s3 and s4).

Analyzing nuclear extracts, the level of the regulating cell cycle transcription factor NF-*κ*B decreases in slower samples, suggesting that the oxidation status into the nuclei leads to destabilization and nuclear export. On the other hand, Nrf2 presence into the nuclei increases from s1 to s4, because Nrf2 acts as a negative regulator of cell cycle entry in hematopoietic stem cells [36].

**Figure 5:**
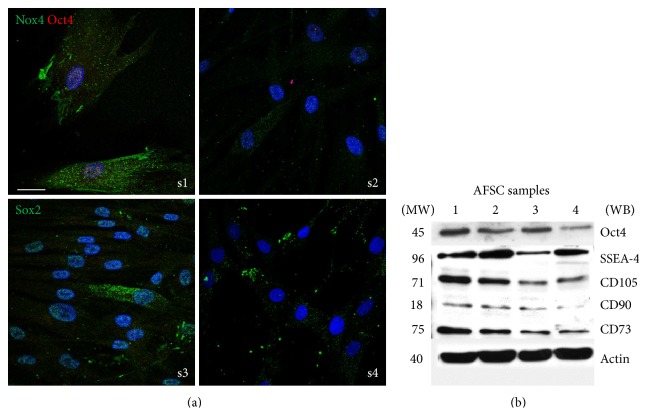
Effect of donor heterogeneity on stem cells markers. (a) Representative images showing superimposing between DAPI (blue), Nox4 (green), and Oct4 (red) signals or DAPI (blue) and Sox2 (green) signals of AFSC samples 1 and 4. Scale bar: 10 *μ*m. (b) Representative images of total lysates of AFSC samples 1–4 separated by SDS-PAGE. Western blot was then performed with the indicated antibodies. Presented data are representative of three independent experiments.

The expression profile of pluripotent stem cells and mesenchymal stem cells markers were analyzed. Figures 5(a) and 5(b) show that nuclear expression markers of pluripotency such as Oct4, Sox2, and SSEA-4 decrease from s1 to s4, as well as the presence of mesenchymal stem cell markers CD73, CD90, and CD105. Therefore the stemness capability could decline.

**Figure 6:**
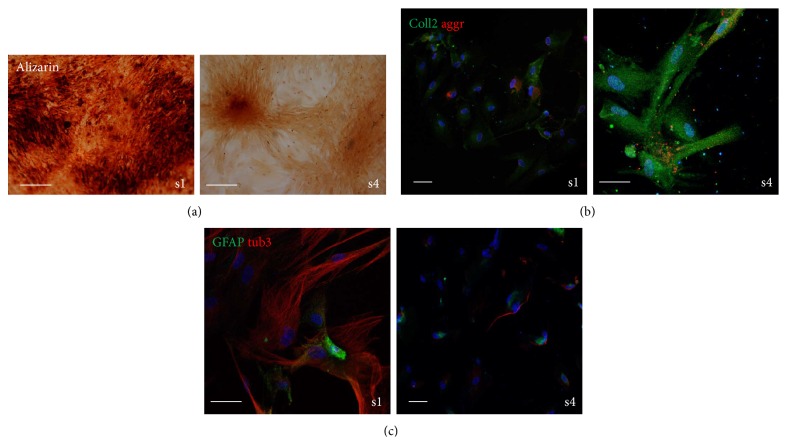
Effect of donor heterogeneity on differentiation potential. (a) Representative images showing staining with alizarin red of AFSC samples 1 and 4 after three weeks of culture in osteogenic medium. (b) Representative images showing superimposing between DAPI (blue), collagen II (green), and aggrecan (red) signals of AFSC samples 1–4 after three weeks of culture in chondrogenic medium. (c) Representative images showing superimposing between DAPI (blue), GFAP (green), and *β*tubulin III (red) signals of AFSC samples 1 and 4 after three weeks of culture in neurogenic medium. Scale bar: 10 *μ*m.

Then we treated AFSC with 3 different differentiation protocols and we tested the presence of calcified matrix (alizarin red) for the osteogenic one, of collagen II and aggrecan for the chondrogenic one, and of GFAP and *β*tubulin III for the neurogenic one. The differentiation potential analysis demonstrated that osteogenic (Figure 6(a)) and neurogenic (Figure 6(c)) differentiations were easier for sample 1 than for sample 4. On the other hand, the presence of cartilage matrix proteins is higher in sample 4 than in sample 1 (Figure 6(b)).

## 4. Discussion

The current effort in regenerative medicine is the use of human stem cells that are easy to collect and are high proliferating, with large plasticity and without ethical problem. Amniotic fluid stem cells show all these characteristics, but there is a donor-to-donor heterogeneity that can influence the proliferation and the differentiation capacities. This is evident starting from the initial phase of culture, before the selection for c-Kit. The difference may be due to the fact that amniotic fluid contains cells of mixed populations derived from fetus and amnion. Nevertheless, this growth difference is maintained also after c-Kit^+^ cells selection. Therefore, it is important to find alternative factors involved in cell fate changes such as ROS and discuss their roles in the pluripotency and the differentiation of stem cells to improve directed culture protocols [3].

Recently, it has become evident that nuclear redox signaling is an important signaling mechanism regulating a variety of cellular functions [37]. NADPH oxidase family (Nox) is one of the most important sources of ROS in several cellular compartments, including the nucleus. Recently, we demonstrated that in AFSC Nox4 can be detected inside the nuclear domains [18]. In the present study we shed light on the type of nuclear domain where Nox4 localizes, namely, speckles domains. Speckles are subnuclear structures that are enriched in premessenger RNA splicing factors and are located in the interchromatin regions of the nucleoplasm of mammalian cells. Speckles are dynamic structures, and both their protein and RNA-protein components can cycle continuously between speckles and other nuclear locations. Several kinases and phosphatases that can regulate the splicing machinery have also been localized into nuclear speckles. They might also contain transcription factors, together with splicing factors [38].

Indeed, transcription factors, as well as even kinases and phosphatases, have been described to be redox regulated in the nucleus, through modulation of their DNA binding capacity [37]. The diversity in transcriptional control is achieved through a complex network of combinatorial protein-protein and protein-DNA interactions affecting the stability and subnuclear localization of these transcriptional regulators. The forkhead homeobox type O (FOXO) transcription factors have an essential role in maintaining stem cell identity [3]. FoxO1, FoxO3a, and FoxO4 are critical mediators of the cellular responses to oxidative stress and can also be viewed as sensors for oxidative stress since their activity is regulated by H_2_O_2_ and, dependent on the cellular context, they relay these stresses to induce apoptosis, stress resistance, or senescence [39, 40]. An increase in intracellular ROS facilitates the localization of FoxO in the nucleus where it is transcriptionally active [40]. Therefore, we investigated at first the localization of FoxO proteins in AFSC expressing Nox4 also into the nuclei. The signal of FoxO1 corresponds with the one of Nox4 but in cytosolic compartment.

Since our interest is to elucidate the role of Nox4 into the nucleus, we examined the nuclear interaction with other transcription factors. For example the transcription factor Oct4 plays essential functions in the maintenance of pluripotent embryonic and germ cells of mammals [41]. Moreover, Oct4 protein has been previously reported to be associated, in human oocytes, with splicing speckles and Cajal bodies [42]. Here we showed that in AFSC nuclei Oct4 colocalizes with Nox4 and sc-35, as speckles marker. Moreover confocal and coimmunoprecipitation analysis demonstrated that Nox4 interacts with speckle domains, suggesting that Nox4 could be involved in the regulation of the transcription/pre-mRNA processing machinery by ROS production in these specific nuclear areas. In fact, immunofluorescent localization of Nox4 demonstrated a punctate pattern of staining in stem cell nuclei, matching with Oct4, a stemness regulating protein. It is possible that Oct4 modulated by Nox4-derived ROS could coordinate with other speckles proteins to regulate RNA processing.

Stem cells isolated from different amniotic fluids exhibit a proliferation rate inversely coupled with Nox4-derived ROS level into the nuclei, as shown by the cell cycle protein analysis. In support of this, there is recently reported evidence that accumulation of oxidative DNA damage restricts the self-renewal capacity of human HSCs [43]. Therefore, we analyzed in different AFSC samples the presence of H2A foci, as marker of DNA damage. As expected, in samples where Nox4 was mostly nuclear, a higher DNA damage occurred. Therefore one potential role of nNox4 could be the regulation of the response to DNA damage or through regulation of DNA repair.

The study of cell cycle better clarified that slower AFSC samples are blocked in G_0_/G_1_ phase. Among the transcription factor, NF-*κ*B and Nrf2 are redox sensitive and cell cycle regulators. In unstimulated cells, NF-*κ*B is sequestered in an inactive form in the cytosol. It can be released from these cytosolic pools by two main pathways (for review, see [44]), resulting in nuclear translocation of NF-*κ*B complexes. In our experimental conditions nNox4 derived-ROS seems to establish a decrease in NF-*κ*B expression also into the nuclei.

Nrf-2 is a transcription factor implicated in the cellular responses to oxidative stress. This heterodimer binds to antioxidant-response elements (AREs) and thereby upregulates numerous genes coding for detoxification enzymes, antioxidants, and the enzymes required for* de novo* GSH synthesis [45]. Interestingly Nrf2 acts as a negative regulator of cell-cycle entry in HSCs, maintaining the balance between HSC quiescence and self-renewal [36]. In effect, Nrf2 level increases in AFSC samples where the cell cycle is blocked. Analyzing the cells culture obtained from different donors, we noticed that also the expression of Oct4 declines in low growth rate samples, as well as Sox2. In fact, increasing evidence suggests that Oct4 does not activate transcription of target genes alone but requires DNA-dependent heterodimerization with another DNA-binding transcription factor, the HMG-box protein Sox2 [41].

As far as concerning the differentiation capability of the different AFSC samples, we noticed that the higher expression of stemness markers (sample 1 or 2) is parallel with an easier differentiation potential towards osteogenic and neurogenic lineages. On the other hand, the chondrogenic commitment was better obtained with AFSC population of sample 4, but this result may be justified from the low oxygen condition that allows this differentiation.

Understanding the possible mechanisms by which ROS influence stem cells' fate may provide insights into how the aging of stem cells could be implicated in diseases of aging [46]. Moreover it may indicate new marker of stemness capability in order to easily discriminate active MSC produced for clinical use, with the final outcome that patients are treated only with effective cells and a waste of public funds is prevented.

Our findings not only show the effects of nuclear Nox4-derived ROS on AFSC, but also suggest the mechanisms involved in the regulation of the proliferation and differentiation capacity. Moreover, targeting increased levels of nuclear ROS associated with nonactive stem cells may reverse their decreased stem capacity, as slight variations in ROS content may have important effects on stem cell fate.

## Abbreviations

(AFSC): Amniotic fluid stem cells
(BSA): Bovine serum albumin
(DABCO): 1,4-Diazabicyclo(2.2.2)octane
(DAPI): 4′,6-Diamidino-2-phenylindole
(DPI): Diphenyleneiodonium
(EDTA): Ethylenediaminetetraacetic acid
(MSCs): Mesenchymal stem cells
(PBS): Phosphate buffered saline
(TBS): Tris-buffered saline
(TxTBS): Triton X-100.
